# Evaluation of the Liver Disease Information in Baidu Encyclopedia and Wikipedia: Longitudinal Study

**DOI:** 10.2196/17680

**Published:** 2021-01-18

**Authors:** Fei Sun, Fuchun Yang, Shusen Zheng

**Affiliations:** 1 Division of Hepatobiliary and Pancreatic Surgery, Department of Surgery The First Affiliated Hospital Zhejiang University School of Medicine Hangzhou China

**Keywords:** Baidu Encyclopedia, Wikipedia, internet, website, liver disease, health information, DISCERN instrument, timeliness

## Abstract

**Background:**

The internet has changed the way of people acquiring health information. Previous studies have shown that Wikipedia is a reasonably reliable medical resource, and it has been ranked higher than other general websites in various search engines. Baidu Encyclopedia is one of the most popular encyclopedia websites in China. However, no studies have shown the quality of the content provided in the Baidu Encyclopedia.

**Objective:**

This study aimed to evaluate the quality of liver disease information provided by Wikipedia (in English) and Baidu Encyclopedia (in Chinese) and to perform a comparison of the quality and timeliness of the articles published in these two encyclopedias. Moreover, a 3-year follow-up study was conducted to compare if the information in both these websites was updated regularly over this period.

**Methods:**

We searched for information on liver diseases by using the International Statistical Classification of Diseases and Related Health Problems 10th Revision Version 2016 codes on Wikipedia (in English) and Baidu Encyclopedia (in Chinese). The quality of the articles was assessed using the DISCERN instrument, which consists of 3 sections. We recorded the latest editing date of the webpages and calculated the date interval to evaluate the update timeliness of these websites.

**Results:**

We found 22 entries on liver diseases in Baidu Encyclopedia and 15 articles in Wikipedia between September 15, 2016, and September 30, 2016, and we found 25 entries in Baidu Encyclopedia and 16 articles in Wikipedia between September 15, 2019, and September 30, 2019. In section 1 of the DISCERN instrument, the mean (SE) scores of Baidu Encyclopedia entries were significantly lower than those of Wikipedia articles. In section 2 and section 3 of the DISCERN instrument, the DISCERN scores of Baidu Encyclopedia entries were lower than those of Wikipedia articles, but the differences were not statistically significant. The total DISCERN scores of Baidu Encyclopedia entries were significantly lower than those of Wikipedia articles. The update interval of the entries in Baidu Encyclopedia was found to be significantly longer than that of the articles in Wikipedia.

**Conclusions:**

This study shows that the quality of articles and the reliability of the research content on liver diseases in Wikipedia are better than those of the entries in Baidu Encyclopedia. However, the quality of the treatment choices provided in both Wikipedia and Baidu Encyclopedia is not satisfactory. Wikipedia is updated more frequently than Baidu Encyclopedia, thereby ensuring that the information presented has the most recent research findings. The findings of our study suggest that in order to find accurate health information, it is important to seek the help of medical professionals instead of looking for a prescription amid the confusing information provided on the internet.

## Introduction

Over 4.5 billion internet users were reported worldwide in June 2019, with 854 million of them being in China [1,2]. Nowadays, the internet has become the primary source of information, and it has changed the way of people acquiring medical and health information. More than 50% of the internet users in the United States of America have been reported to search for web-based health care–related information [3,4]. A study in China found that 87.8% of the patients with scoliosis searched for scoliosis-related information on the internet, thereby indicating the high proportion of internet usage as a source of health information [5]. Increasing number of patients are seeking information about their diseases on the internet, but the reliability of web-based health care–related information is still questionable [6,7].

Wikipedia is a web-based encyclopedia that provides valuable web-based health information; it contains more than 5,956,750 articles in English [8-10]. Previous studies have shown that Wikipedia is a reasonably reliable medical resource and it was ranked higher than other general websites in search engines [8,11], although there are some errors in Wikipedia articles compared to peer-reviewed sources [12]. Baidu Encyclopedia, Wikipedia’s equivalent in China, contains more than 16,244,000 entries in Chinese [13]. Wikipedia and Baidu Encyclopedia are the most popular and consulted encyclopedia websites in English and Chinese, respectively [14-16]. On December 9, 2012, Baidu Encyclopedia announced the “rainbow plan,” wherein all medical entries could only be edited and revised by certified medical experts, which would improve the quality of the health information provided in Baidu Encyclopedia [17]. However, no study has yet examined the quality of the data provided in Baidu Encyclopedia.

Liver disease is among the top 10 causes of death in middle-income and high-income countries [18]. We have been engaged in hepatic surgery for many years and we are competent in the diagnosis and treatment of liver diseases. Therefore, we selected liver diseases as the object of analysis in this study. This study aimed to evaluate the quality of liver disease information provided by Wikipedia in English and Baidu Encyclopedia in Chinese and we aimed to perform a comparison of the quality of the research in these resources and the timeliness of the recent updates in the research between these 2 resources. Since the update frequency on the internet is high by day and by hour, 3 years can be considered as an extended period for evaluating the changes in the information over a long interval during follow-up. Thus, 3-year monitoring was conducted to compare whether the information on these websites was improved over this period. Our research results will help readers judge the reliability of web-based encyclopedia entries and avoid the medical problems caused by believing unreliable materials on the internet.

## Methods

### Data Sources

The articles analyzed in this study are available in Wikipedia [9] in English and in Baidu Encyclopedia [13] in Chinese. Data were compiled between September 15, 2016, and September 30, 2016. For the 3-year follow-up, data were compiled between September 15, 2019, and September 30, 2019. We chose 3 years for the length of the comparison because 3 years is a widely used and acceptable follow-up time [19-21].

### Retrieval of Liver Disease Articles

The selection of topics was based on the International Statistical Classification of Diseases and Related Health Problems 10th Revision (ICD-10) version 2016. ICD defines almost all of the health-related conditions, and it is the diagnostic classification standard for all clinical and research purposes. In the ICD-10 version 2016, diseases of the liver are classiﬁed under Chapter XI Diseases of the digestive system, with categories K70 to K77 [22]. The Baidu Encyclopedia (in Chinese) and Wikipedia (in English) were investigated for articles on ICD-10 version 2016 codes. All ICD-10 code titles on the diseases of the liver were used for the search. Entries without search results were excluded from the study. Search terms on Baidu Encyclopedia (in Chinese) and Wikipedia (in English) are listed in the Multimedia Appendices 1 and 2, respectively. The proportion of the available search results were recorded and analyzed.

### Assessment of the Quality of the Research Articles

The quality of the articles was assessed using the DISCERN instrument, which is used for judging the quality of health information on treatment choices [23]. The use of the DISCERN instrument for information in encyclopedias is controversial. Although some studies believe that the original edition of the DISCERN instrument is not suitable for evaluating Wikipedia articles [24,25], there are still many recent studies that have used the DISCERN instrument to evaluate the quality of Wikipedia articles [15,16]. Thus, the DISCERN instrument can be considered as an appropriate tool for evaluating the quality of Wikipedia articles in the absence of a better instrument.

The DISCERN instrument consists of 16 questions that are rated on a 5-point scale (1=definitely no, 5=definitely yes) (Multimedia Appendix 3). All these 16 questions are categorized into 3 sections. Section 1 (questions 1 to 8) assesses the reliability of a paper, section 2 (questions 9 to 15) focuses on the quality of the treatment information, and section 3 (question 16) evaluates the overall quality [23]. A higher DISCERN score indicates better paper quality. The overall DISCERN score ranges from 16 to 80 and the articles were categorized as very poor (16-26), poor (27-38), fair (39-50), good (51-62), and excellent (63-80) based on the scores [15,16].

All 3 authors have been engaged in hepatobiliary and pancreatic surgery for many years and are competent in the diagnosis and treatment of liver diseases. We have mastered the professional knowledge of liver diseases and can make professional evaluations. Sun and Yang used the DISCERN instrument to evaluate the articles in Baidu Encyclopedia (in Chinese) and Wikipedia (in English), respectively. The DISCERN score was determined through discussions. If no agreement was reached during the debate, Zheng was consulted to make the final judgment. Subsequently, Sun and Yang agreed on all the ratings.

### Timeliness of the Updated Articles

We recorded and analyzed the last editing date of the web page. In 2016, the update interval was calculated as the date interval between the updated date and September 20, 2016. In 2019, the update interval was calculated as the date interval between the updated date and September 20, 2019. The update interval reflects the update frequency of a webpage. We compared the update interval of each paper to evaluate the update timeliness of the website.

### Statistical Analysis

For the statistical analysis, means and standard errors (mean [SE]) were calculated. *P* values less than .05 were considered signiﬁcant. Differences between groups were assessed using two-tailed Student *t* test with Welch correction. Statistical analyses were performed using GraphPad Prism 6 software (GraphPad Prism Software Inc).

## Results

### Retrieval of Liver Disease Articles

We searched Baidu Encyclopedia and Wikipedia with the entries in the ICD-10 diseases of the liver category. A total of 8 liver disease categories were classiﬁed, with categories K70 to K77. Only a portion of the entries were retrieved as articles in the search results. We found 22 entries in Baidu Encyclopedia and 15 articles in Wikipedia in 2016 and 25 entries in Baidu Encyclopedia and 16 articles in Wikipedia in 2019 (Table 1). In general, we were able to retrieve more entries in Baidu Encyclopedia than in Wikipedia.

**Table 1:** The proportion of the available search results in Baidu Encyclopedia and Wikipedia in 2016 and 2019.

ICD-10^a^ category	Entries included in the ICD-10 classification (n=50)	Baidu Encyclopedia entries in 2016 (n=22)	Wikipedia articles in 2016 (n=15)	Baidu Encyclopedia entries in 2019 (n=25)	Wikipedia articles in 2019 (n=16)
K70 Alcoholic liver disease	6	4	3	4	3
K71 Toxic liver disease	10	1	1	1	1
K72 Hepatic failure, not elsewhere classified	3	1	1	2	1
K73 Chronic hepatitis, not elsewhere classified	5	3	1	3	1
K74 Fibrosis and cirrhosis of liver	7	4	1	5	1
K75 Other inflammatory liver diseases	7	3	2	3	2
K76 Other diseases of liver	10	6	6	7	7
K77 Liver disorders in diseases classified elsewhere	2	0	0	0	0

^a^ICD-10: International Statistical Classification of Diseases and Related Health Problems 10th Revision.

### Assessment of the Quality of Articles

**Figure 1:**
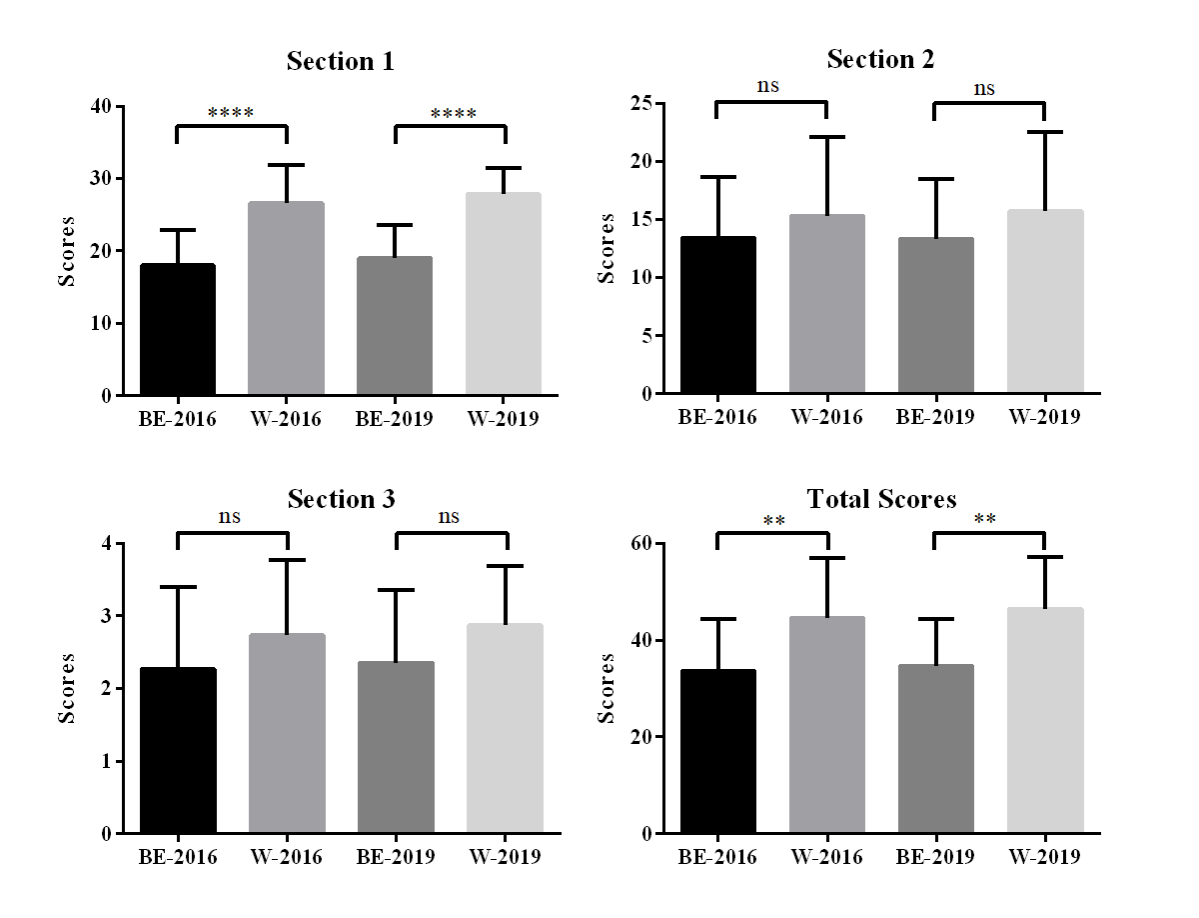
The DISCERN scores of Baidu Encyclopedia entries and Wikipedia articles on liver diseases in 2016 and 2019. **P<.01, ****P<.001. BE-2016: Baidu Encyclopedia entries in 2016; BE-2019: Baidu Encyclopedia entries in 2019; W-2016: Wikipedia articles in 2016; W-2019: Wikipedia articles in 2019; ns: no significant difference.

We used the DISCERN instrument to score the articles retrieved in Baidu Encyclopedia and Wikipedia and compared the section 1, section 2, section 3, and total scores (Figure 1). The results of the DISCERN instrument were comparable in 2016 and 2019. In section 1, the mean (SE) DISCERN score of Baidu Encyclopedia entries was significantly lower than that of Wikipedia articles (18.00 [SE 1.040] vs 26.60 [SE 1.359], *P*<.001 in 2016, respectively; 19.00 [SE 0.9110] vs 27.88 [SE 0.8892], *P*<.001 in 2019, respectively). In section 2 and section 3 of the DISCERN instrument, the mean DISCERN scores of Baidu Encyclopedia entries were lower than those of Wikipedia articles, but the differences were not statistically significant. Overall, the total scores of Baidu Encyclopedia entries were significantly lower than those of Wikipedia articles (33.68 [SE 2.265] vs 44.67 [SE 3.172], *P*=.009 in 2016, respectively; 34.72 [SE 1.943] vs 46.44 [SE 2.669], *P*=.001 in 2019, respectively) (Table 2).

The total DISCERN scores were categorized. In Baidu Encyclopedia in 2016, 9 entries were found to be of fair quality, 8 were of poor quality, and 8 were of very poor quality. In 2019, 10 entries were of fair quality, 11 were of poor quality, and 4 were of very poor quality. In Wikipedia in 2016, 5 articles were of good quality, 5 were of fair quality, 3 were of poor quality, and 2 were found to be of very poor quality. In 2019, 6 articles were found to be of good quality, 4 were of fair quality, and 5 were of poor quality.

Over time, the number of entries in Baidu Encyclopedia and Wikipedia showed an increase (Table 1). However, no significant differences were found on comparing the changes in the DISCERN scores of Baidu and Wikipedia entries over 3 years.

**Table 2:** The DISCERN scores of Baidu Encyclopedia and Wikipedia articles on liver diseases.

Year, sections of DISCERN		Baidu Encyclopedia, mean (SE)	Wikipedia, mean (SE)	*P* value
**2016**				
	Section 1	18.00 (1.040)	26.60 (1.359)	<.001
	Section 2	13.36 (1.023)	15.69 (1.717)	.25
	Section 3	2.273 (0.2389)	2.733 (0.2667)	.21
	Total	33.68 (2.265)	44.67 (3.172)	.009
**2019**				
	Section 1	19.00 (0.9110)	27.88 (0.8892)	<.001
	Section 2	13.36 (1.023)	15.69 (1.717)	.25
	Section 3	2.360 (0.1990)	2.875 (0.2016)	.07
	Total	34.72 (1.943)	46.44 (2.669)	.001

### Timeliness of Articles

**Figure 2:**
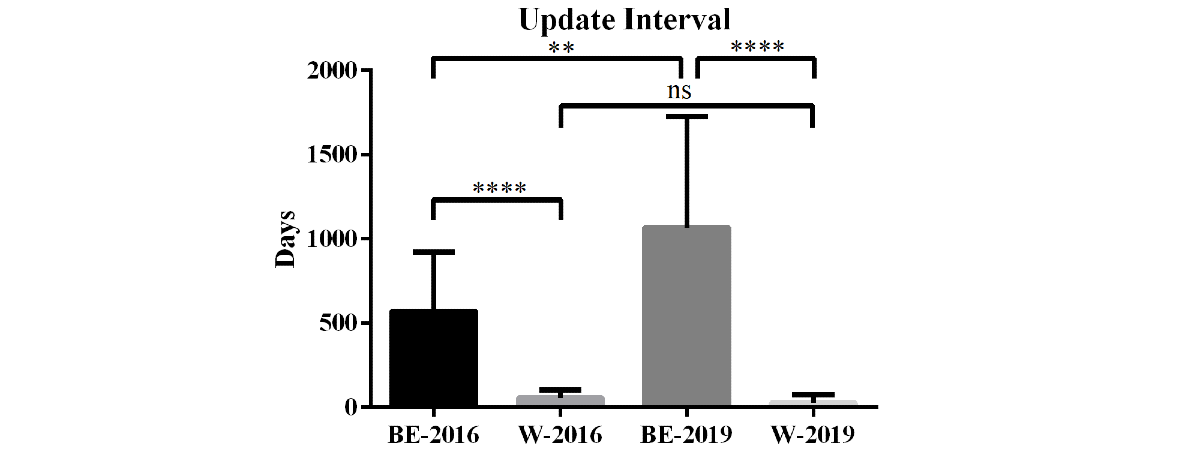
The update interval of Baidu Encyclopedia entries and Wikipedia articles on liver diseases in 2016 and 2019. **P<.01, ****P<.001. BE-2016: Baidu Encyclopedia entries in 2016; BE-2019: Baidu Encyclopedia entries in 2019; W-2016: Wikipedia articles in 2016; W-2019: Wikipedia articles in 2019; ns: no significant difference.

The update interval represents the timeliness of the updates on the website. We compared the update interval between Baidu Encyclopedia and Wikipedia (Figure 2). In 2016, the mean (SE) update interval of Baidu Encyclopedia entries was 571.6 (74.96) days and the mean (SE) update interval of Wikipedia entries was 55.93 (11.92) days. The update interval for entries in Baidu Encyclopedia was significantly longer than that for articles in Wikipedia (*P*<.001). After 3 years of follow-up, it was found that 11 of the 25 entries in Baidu Encyclopedia were recently edited before September 20, 2016, which meant that they had not been updated in the last 3 years and were in a state of loss of maintenance. In 2019, the mean (SE) update interval of Baidu Encyclopedia entries was 1067 (131.2) days and the mean (SE) update interval of Wikipedia articles was 27.44 (11.98) days. The update interval of the entries in Baidu Encyclopedia is significantly longer than that of the articles in Wikipedia (*P*<.001) (Figure 2). Therefore, the update timeliness of the articles in Wikipedia in 2016 and 2019 is significantly better than that of the entries in Baidu Encyclopedia.

## Discussion

### Principal Findings

The main findings of this study were that the reliability of the articles on Wikipedia is better than that of the entries in Baidu Encyclopedia. However, the quality of the treatment information in both the encyclopedia websites is not satisfactory. With the increasing popularity of the internet, increasing number of patients are seeking health information and even treatment plans for their diseases on the internet [4]. However, the quality of the medical information on the internet is uneven, and a large proportion of the information is unreliable [6]. Previous research has shown that health information in a search engine is less reliable [26-28]. Wikipedia is a remarkable source of web-based health information [8]. Therefore, we analyzed the information on liver diseases in Baidu Encyclopedia in Chinese and Wikipedia in English to compare the quality of the medical information in these encyclopedia websites. The quality of the health information on the internet was studied over a 3-year follow-up period to see if it improved over time.

We evaluated the articles on liver diseases in Baidu Encyclopedia and Wikipedia in terms of quantity and quality. The Baidu Encyclopedia has more overall entries than Wikipedia; therefore, it is reasonable for Baidu Encyclopedia to have more entries in the category of liver disease than Wikipedia. In Wikipedia, terms can be easily linked to each other, making it easier for readers to build connections of knowledge [29,30]. In Baidu Encyclopedia, although there are abundant entries, the isolated entries are not linked with the other related entries, thereby making it unconducive to the formation of knowledge networks for some concepts.

We used the DISCERN instrument to evaluate the quality and reliability of the articles in Baidu Encyclopedia and Wikipedia. The DISCERN scores of Wikipedia entries were significantly higher than those of Baidu Encyclopedia entries. Consistent with that reported in previous studies, Wikipedia is a good resource of health information on the internet [8,14]. In section 1 of the DISCERN instrument, which reflects the reliability of articles, the scores for the entries in Baidu Encyclopedia were significantly lower than those for the articles in Wikipedia, indicating that the entries in Baidu Encyclopedia were less reliable than those in Wikipedia. We observed that most of the Baidu Encyclopedia entries lacked reference sources, while Wikipedia includes a detailed source for almost every entry. A high-quality citation source is an essential guarantee for the reliability of a paper [31]. However, several studies have found that Wikipedia is difficult for the general public to read and is not a reliable source for medical students [25,32]. During the 2019 follow-up, we observed the source of citations for each paper. The sources of Baidu Encyclopedia references were found to be ambiguous; they were mostly search results of Baidu academic search (an academic search engine of Baidu) and did not represent the literature cited by the entry. However, citations in Wikipedia can be explicitly identified and have original links to academic databases such as PubMed. Accurate referencing is crucial for improving the authoritativeness and credibility of a paper [31,33]. In this regard, Wikipedia was found to be better than Baidu Encyclopedia, as the basic ideas are cited and most of the references are academic papers, which guarantee scientific accuracy and reliability. In section 2 of the DISCERN instrument, which assesses the quality of the information on treatment choices, the average score of the articles in Wikipedia was found to be higher than that of the entries in Baidu Encyclopedia. However, the difference between them was not statistically significant. The scores of both Wikipedia and Baidu Encyclopedia entries were low in section 2. The quality of the information on treatment choices in both Wikipedia and Baidu Encyclopedia was not satisfactory, which is in accordance with that reported in previous studies [16,25,32]. After all, Wikipedia and Baidu Encyclopedia are not professional medical websites. Both Wikipedia and Baidu Encyclopedia have room for further improvement in providing information on treatment. Since the choice of treatment is an important target for patients to search for, adequate information should also be provided for the choice of treatment. References to international guidelines for various diseases and attracting, encouraging, and even recruiting more medical professionals to participate in the editing of the medical information provided in these websites can improve the quality of the information provided in these websites. Further, patients should be advised to seek treatment advice from more professional medical websites, and it would be more reliable to seek professional advice directly from medical personnel [34]. In section 3 of the DISCERN instrument, the score for Baidu Encyclopedia entries was lower than that for Wikipedia articles, which is consistent with the evaluation result of the total score.

In terms of the update timeliness, Wikipedia was significantly better than Baidu Encyclopedia in 2016 and 2019. During the 3-year follow-up, all Wikipedia pages were updated on time. As for Baidu Encyclopedia, among the 25 entries, 11 entries were not updated in 3 years and the longest update time reached 2564 days. Wikipedia is updated regularly and it evolves continuously while the content in Baidu Encyclopedia has been rarely maintained since its generation. The low update frequency has caused some information to be outdated, and some outdated information may even be wrong. Therefore, the timeliness of information is also an important aspect influencing the quality of medical information.

Our study uses the DISCERN instrument to evaluate the quality of Baidu Encyclopedia entries and compares the quality of these entries with that of Wikipedia articles. This is the first study to assess the quality of health information in Baidu Encyclopedia with the DISCERN instrument. Moreover, this is the first study to follow up on the quality of encyclopedia websites for 3 years. In 3 years, we found that the quality of the articles of both websites had not improved significantly.

### Limitations

Our study design does have some limitations. First, the results of our research only reflect the information on liver diseases in Baidu Encyclopedia and Wikipedia on September 20, 2016, and September 20, 2019. Second, the DISCERN instrument is the only tool used to evaluate article quality in this study. Although the DISCERN instrument is widely used to evaluate website information, comprehensive use of more evaluation tools may make the results more convincible. The LIDA tool is a web-based validation instrument to measure the design and content of health information on the internet [35]. We plan to use the LIDA tool to evaluate the quality of articles in Baidu Encyclopedia and Wikipedia in our future research.

### Conclusion

Although Wikipedia is not as good as the Baidu Encyclopedia in terms of the number of entries, its reliability is better than that of Baidu Encyclopedia, thereby making it more helpful to web-based health information seekers. However, the quality of the treatment choices in both Wikipedia and Baidu Encyclopedia’s entries is not satisfactory. Wikipedia is updated frequently to keep the information up-to-date. The entries in Baidu Encyclopedia lack maintenance and are not updated on time; some information is outdated and some new content are lacking. In order to find accurate health information, people are advised to seek the help of medical professionals instead of looking for a prescription amid the confusing information on the internet.

## Appendix

Multimedia Appendix 1 International Statistical Classification of Diseases and Related Health Problems 10th Revision code search terms for liver diseases in Baidu Encyclopedia in 2019.

Multimedia Appendix 2 International Statistical Classification of Diseases and Related Health Problems 10th Revision code search terms for liver diseases in Wikipedia in 2019.

Multimedia Appendix 3 The DISCERN instrument.

## Abbreviations

ICD-10: International Statistical Classification of Diseases and Related Health Problems 10th Revision
