# Supplementary material for: Evaluation of the Liver Disease Information in Baidu Encyclopedia and Wikipedia: Longitudinal Study
Source: J Med Internet Res. 2021 Jan 18;23(1):e17680. doi: 10.2196/17680 (PMC7850904; doi:10.2196/17680)
Supplement: Multimedia Appendix 1 [file jmir_v23i1e17680_app1.pdf]

ICD-10	Search Terms in Chinese	URL	DISCERN score			
			Section1	Section2	Section3	Total Score
K70.0 Alcoholic fatty liver	酒精性脂肪肝	https://baike.baidu.com/item/%E9%85%92%E7%B2%BE%E6%80%A7%E8%84%82%E8%82%AA%E8%82%9D	24	17	3	44
K70.1 Alcoholic hepatitis	酒精性肝炎	https://baike.baidu.com/item/%E9%85%92%E7%B2%BE%E6%80%A7%E8%82%9D%E7%82%8E	20	18	3	41
K70.3 Alcoholic cirrhosis of liver	酒精性肝硬化	https://baike.baidu.com/item/%E9%85%92%E7%B2%BE%E6%80%A7%E8%82%9D%E7%A1%AC%E5%8C%96	19	10	2	31
K70.9 Alcoholic liver disease, unspecified	酒精性肝病	https://baike.baidu.com/item/%E9%85%92%E7%B2%BE%E6%80%A7%E8%82%9D%E7%97%85	26	14	3	43
K71.9 Toxic liver disease, unspecified	中毒性肝病	https://baike.baidu.com/item/%E4%B8%AD%E6%AF%92%E6%80%A7%E8%82%9D%E7%97%85	10	7	1	18
K72.1 Chronic hepatic failure	慢性肝衰竭	https://baike.baidu.com/item/%E6%85%A2%E6%80%A7%E8%82%9D%E8%A1%B0%E7%AB%AD	20	9	2	31
K72.9 Hepatic failure, unspecified	肝衰竭	https://baike.baidu.com/item/%E8%82%9D%E8%A1%B0%E7%AB%AD	25	22	3	50
K73.0 Chronic persistent hepatitis, not elsewhere classified	慢性持续性肝炎	https://baike.baidu.com/item/%E6%85%A2%E6%80%A7%E6%8C%81%E7%BB%AD%E6%80%A7%E8%82%9D%E7%82%8E	16	11	1	28
K73.2 Chronic active hepatitis, not elsewhere classified	慢性活动性肝炎	https://baike.baidu.com/item/%E6%85%A2%E6%80%A7%E6%B4%BB%E5%8A%A8%E6%80%A7%E8%82%9D%E7%82%8E	9	7	1	17

K73.9 Chronic hepatitis, unspecified	慢性肝炎	https://baike.baidu.com/item/%E6%85%A2%E6%80%A7%E8%82%9D%E7%82%8E	23	20	4	47
K74.0 Hepatic fibrosis	肝纤维化	https://baike.baidu.com/item/%E8%82%9D%E7%BA%A4%E7%BB%B4%E5%8C%96	22	11	2	35
K74.1 Hepatic sclerosis	肝硬化	https://baike.baidu.com/item/%E8%82%9D%E7%A1%AC%E5%8C%96	26	20	4	50
K74.3 Primary biliary cirrhosis	原发性胆汁性肝硬化	https://baike.baidu.com/item/%E5%8E%9F%E5%8F%91%E6%80%A7%E8%83%86%E6%B1%81%E6%80%A7%E8%82%9D%E7%A1%AC%E5%8C%96	16	12	1	29
K74.4 Secondary biliary cirrhosis	继发性胆汁性肝硬化	https://baike.baidu.com/item/%E7%BB%A7%E5%8F%91%E6%80%A7%E8%83%86%E6%B1%81%E6%80%A7%E8%82%9D%E7%A1%AC%E5%8C%96	17	11	2	30
K74.5 Biliary cirrhosis, unspecified	胆汁性肝硬化	https://baike.baidu.com/item/%E8%83%86%E6%B1%81%E6%80%A7%E8%82%9D%E7%A1%AC%E5%8C%9	19	20	3	42
K75.0 Abscess of liver	肝脓肿	https://baike.baidu.com/item/%E8%82%9D%E8%84%93%E8%82%BF	18	14	2	34
K75.3 Granulomatous hepatitis, not elsewhere classified	肉芽肿性肝病	https://baike.baidu.com/item/%E8%82%89%E8%8A%BD%E8%82%BF%E6%80%A7%E8%82%9D%E7%97%85/1619029	17	7	1	25
K75.4 Autoimmune hepatitis	自身免疫性肝炎	https://baike.baidu.com/item/%E8%87%AA%E8%BA%AB%E5%85%8D%E7%96%AB%E6%80%A7%E8%82%9D%E7%82%8E	24	21	4	49
K76.0 Fatty (change of) liver, not elsewhere classified	肝脂肪变性	https://baike.baidu.com/item/%E8%82%9D%E8%84%82%E8%82%AA%E5%8F%98%E6%80%A7/10968800	11	7	1	19

K76.3 Infarction of liver	肝梗死	https://baike.baidu.com/item/%E8%82%9D%E6%A2%97%E6%AD%BB	17	9	2	28
K76.4 Peliosis hepatis	肝紫癜病	https://baike.baidu.com/item/%E8%82%9D%E7%B4%AB%E7%99%9C%E7%97%85/2523336	18	10	3	31
K76.5 Hepatic veno-occlusive disease	肝静脉闭塞病	https://baike.baidu.com/item/%E8%82%9D%E9%9D%99%E8%84%89%E9%97%AD%E5%A1%9E%E7%97%85/14738785	20	14	3	37
K76.6 Portal hypertension	门静脉高压症	https://baike.baidu.com/item/%E9%97%A8%E9%9D%99%E8%84%89%E9%AB%98%E5%8E%8B%E7%97%	17	19	3	39
K76.7 Hepatorenal syndrome	肝肾综合征	https://baike.baidu.com/item/%E8%82%9D%E8%82%BE%E7%BB%BC%E5%90%88%E5%BE%81	20	17	3	40
K76.9 Liver disease, unspecified	肝脏疾病	https://baike.baidu.com/item/%E8%82%9D%E8%84%8F%E7%96%BE%E7%97%85	21	7	2	30
