# Supplementary material for: Evaluation of the Liver Disease Information in Baidu Encyclopedia and Wikipedia: Longitudinal Study
Source: J Med Internet Res. 2021 Jan 18;23(1):e17680. doi: 10.2196/17680 (PMC7850904; doi:10.2196/17680)
Supplement: Multimedia Appendix 2 [file jmir_v23i1e17680_app2.pdf]

ICD-10	Search Terms	URL	DISCERN score			
			Section1	Section2	Section3	Total Score
K70.1 Alcoholic hepatitis	Alcoholic hepatitis	https://en.wikipedia.org/wiki/Alcoholic_hepatitis	30	19	4	53
K70.3 Alcoholic cirrhosis of liver	Cirrhosis	https://en.wikipedia.org/wiki/Cirrhosis	31	25	4	60
K70.9 Alcoholic liver disease, unspecified	Alcoholic liver disease	https://en.wikipedia.org/wiki/Alcoholic_liver_disease	29	21	3	53
K71.9 Toxic liver disease, unspecified	Hepatotoxicity	https://en.wikipedia.org/wiki/Hepatotoxicity	31	12	3	46
K72.9 Hepatic failure, unspecified	Liver failure	https://en.wikipedia.org/wiki/Liver_failure	22	7	2	31
K73.9 Chronic hepatitis, unspecified	Hepatitis	https://en.wikipedia.org/wiki/Hepatitis#Causes	33	26	4	63
K74.3 Primary biliary cirrhosis	Primary biliary cholangitis	https://en.wikipedia.org/wiki/Primary_biliary_cholangitis	33	24	3	60
K75.0 Abscess of liver	Liver abscess	https://en.wikipedia.org/wiki/Liver_abscess	21	9	2	32
K75.4 Autoimmune hepatitis	Autoimmune hepatitis	https://en.wikipedia.org/wiki/Autoimmune_hepatitis	25	10	2	37
K76.0 Fatty (change of) liver, not elsewhere classified	Fatty liver disease	https://en.wikipedia.org/wiki/Fatty_liver_disease	30	16	3	49
K76.1 Chronic passive congestion of liver	Congestive hepatopathy	https://en.wikipedia.org/wiki/Congestive_hepatopathy	25	8	2	35
K76.4 Peliosis hepatis	Peliosis hepatis	https://en.wikipedia.org/wiki/Peliosis_hepatis	26	9	2	37
K76.5 Hepatic veno-occlusive disease	Hepatic veno-occlusive disease	https://en.wikipedia.org/wiki/Hepatic_veno-occlusive_disease	27	11	2	40
K76.6 Portal hypertension	Portal hypertension	https://en.wikipedia.org/wiki/Portal_hypertension	28	21	3	52
K76.7 Hepatorenal syndrome	Hepatorenal syndrome	https://en.wikipedia.org/wiki/Hepatorenal_syndrome	29	23	4	56
K76.9 Liver disease, unspecified	Liver disease	https://en.wikipedia.org/wiki/Liver_disease	26	10	3	39
