# Supplementary material for: Evaluation of the Liver Disease Information in Baidu Encyclopedia and Wikipedia: Longitudinal Study
Source: J Med Internet Res. 2021 Jan 18;23(1):e17680. doi: 10.2196/17680 (PMC7850904; doi:10.2196/17680)
Supplement: Multimedia Appendix 3 [file jmir_v23i1e17680_app3.doc]

Multimedia Appendix

| *The DISCERN instrument* |
| --- |
| *Section 1: Is the publication reliable?* |
| Question 1: Are the aims clear? |
| Question 2: Does it achieve its aims? |
| Question 3: Is it relevant? |
| Question 4: Is it clear what sources of information were used to compile the publication (other than the author or producer)? |
| Question 5: Is it clear when the information used or reported in the publication was produced? |
| Question 6: Is it balanced and unbiased? |
| Question 7: Does it provide details of additional sources of support and information? |
| Question 8: Does it refer to areas of uncertainty? |
| *Section 2: How good is the quality of information on treatment choices?* |
| Question 9: Does it describe how each treatment works? |
| Question 10: Does it describe the benefits of each treatment? |
| Question 11: Does it describe the risks of each treatment? |
| Question 12: Does it describe what would happen if no treatment is used? |
| Question 13: Does it describe how the treatment choices affect overall quality of life? |
| Question 14: Is it clear that there may be more than one possible treatment choice? |
| Question 15: Does it provide support for shared decision-making? |
| *Section 3: Overall rating* |
| Question 16: Based on the answers to all of the above questions, rate the overall quality of the publication as a source of information about treatment choices. |

This is a Multimedia Appendix to a full manuscript published in the J Med Internet Res. For full copyright and citation information see http://dx.doi.org/10.2196/jmir.17680
